# Accuracy of nanopore-based targeted next-generation sequencing assay for detection of *Mycobacterium tuberculosis* and drug resistance from non-sputum specimens: a multicenter prospective study in China

**DOI:** 10.1128/jcm.01433-25

**Published:** 2026-02-24

**Authors:** Zeliang Yang, Zichun Ma, Zubi Liu, Peibo Li, Yuqin Liu, Long Cai, Biyi Su, Dan Li, Lele Wang, Lu Cui, Rui Shao, Dapeng Fan, Yaoju Tan, Dan Shen, Hong Shi, Liang Li, Yu Pang

**Affiliations:** 1 Beijing Key Laboratory for Key Technologies in Tuberculosis Prevention and Control, Department of Bacteriology and Immunology, Beijing Chest Hospital, Capital Medical University/Beijing Tuberculosis and Thoracic Tumor Research Institute 12517, Beijing, China; 2 Zhejiang Key Laboratory of Digital Technology in Medical Diagnostics, Dian Diagnostics Group Co., Ltd. 654498 , Hangzhou, Zhejiang, China; 3 Department of Infectious Diseases, Sir Run Run Shaw Hospital of Zhejiang University School of Medicine 26441, Hangzhou, China; 4 Department of the Fifth Tuberculosis, Chongqing Public Health Medical Center 567508, Chongqing, China; 5 Infectious Disease Hospital of Heilongjiang Province 678485 , Harbin, Heilongjiang, China; 6 Department of Clinical Laboratory, Hangzhou Red Cross Hospital 117871, Hangzhou, China; 7 Department of Clinical Laboratory, Guangzhou Chest Hospital, Guangzhou, China; The University of North Carolina at Chapel Hill School of Medicine, Chapel Hill, North Carolina, USA

**Keywords:** tuberculosis, non-sputum specimens, nanopore-based targeted next-generation sequencing, drug susceptibility, diagnosis

## Abstract

Tuberculosis (TB) remains the leading cause of mortality caused by a single infectious agent. Targeted next-generation sequencing (tNGS) has become a promising molecular method for TB diagnosis, but its accuracy and application for non-sputum specimens still remain unexplored. In this multicenter prospective lab-developed assay, 701 participants from five designated TB hospitals from different provinces in China were recruited. Non-sputum specimens were collected for tNGS, Xpert *Mycobacterium tuberculosis* (MTB)/RIF (Xpert), and culture tests. The diagnostic accuracy of tNGS for TB patients was evaluated compared with the microbiological reference standard (MRS). Phenotypic drug susceptibility tests (DSTs) were conducted using culture-positive isolates and employed to assess the accuracy of tNGS for detecting drug resistance. We found that the tNGS assay exhibited high diagnostic accuracy with sensitivity and specificity of 93.4% (95% CI, 91.5%–95.2%) and 93.2% (95% CI, 91.3%–95.0%) when utilizing MRS as the gold standard. The diagnostic performance of tNGS was robust regardless of specimen types and clinical symptoms. tNGS also showed a detection potential for other coinfecting respiratory pathogens. More than 90% of tNGS-positive individuals gained drug susceptibility results, which were mainly dependent on bacterial loads. Compared with phenotypical DSTs, tNGS had high sensitivities and specificities for the detection of rifampicin, isoniazid, streptomycin, ethambutol, and levofloxacin resistance. Our findings illustrated that tNGS assay is a rapid and highly sensitive test for TB diagnosis and simultaneous detection of drug resistance in non-sputum specimens. Its accuracy gain compared with conventional methods is most remarkable in TB patients with low bacterial loads.

**IMPORTANCE:**

Tuberculosis (TB) diagnosis still remains challenging, especially in non-sputum patients. Nanopore-based targeted next-generation sequencing (tNGS) is a promising technology for the detection of TB cases and drug resistance, which has capacities to provide a panel of drug resistance profiles. The purpose of this study is to explore the diagnostic performance of tNGS for non-sputum specimens from five designated TB hospitals from different regions in China. Compared with the microbiological reference standard (MRS), tNGS exhibited high sensitivity and specificity, which are associated with bacterial loads in samples. Meanwhile, tNGS has capacities to detect coinfecting respiratory pathogens and produce drug-resistant profiles. Therefore, our findings suggested that tNGS is an alternative method for the diagnosis of non-sputum TB patients.

**CLINICAL TRIALS:**

This study is registered with Chinese Clinical Trial Registry as ChiCTR2400088518.

## INTRODUCTION

Tuberculosis (TB), caused by *Mycobacterium tuberculosis* (MTB), still remains a major public health challenge. In 2023, more than 10.8 million people developed TB, with 1.3 million deaths from TB worldwide (1). Most importantly, the SARS-CoV-2 pandemic abruptly reversed the declining trend in TB incidence, and a substantial reduction in TB testing and case notification has taken global control progress back by 10 years (2, 3). Approximately 52% of patients are missed by local healthcare systems annually, leading to the ongoing transmission within communities (1). Early and accurate diagnosis of infectious TB cases is therefore crucial to advancing global efforts toward achieving the target of ending tuberculosis by 2035.

Conventional diagnostic tests for TB have limitations that impede the early identification of this disease (4). Sputum smear microscopy exhibits relatively low sensitivity and fails to detect patients with paucibacillary TB (5). Mycobacterial culture, the current gold standard, requires up to 2 months to yield results. Recently, nucleic acid amplification testing (NAAT) has facilitated early case detection, thus curbing disease spread and enabling the prompt initiation of healthcare. The World Health Organization (WHO) has endorsed multiple NAATs to detect active TB and drug-resistant TB strains (6). Despite providing shorter assay turnaround times compared to culture, PCR-based amplification cannot achieve simultaneous detection of multiple targets due to the limited number of fluorescent channels (7, 8). Thus, there is an urgent need to develop novel molecular diagnostic technologies to address this current dilemma, such as those capable of generating large volumes of next-generation sequencing data (9).

In recent years, increasing attention has been devoted to the application of next-generation sequencing for species identification and drug-resistance genotyping of mycobacteria (10). Nanopore sequencing offers rapid real-time sequencing capabilities and ultra-long reads, providing potential for clinical diagnosis of bacterial lower respiratory tract infections (11, 12). It also exhibits sensitivity comparable to, or even higher than, the existing culture-based gold standard method. Despite these impressive advantages, knowledge about the diagnostic performance of nanopore sequencing on active TB cases remains limited, especially regarding non-sputum specimens (13–15). To address this concern, we performed a multicenter prospective study in China to determine the accuracy of nanopore-based targeted next-generation sequencing (tNGS) assay for the detection of MTB and drug resistance from multiple non-sputum specimens (Graphical abstract).

## MATERIALS AND METHODS

### Study design

**Fig 1:**
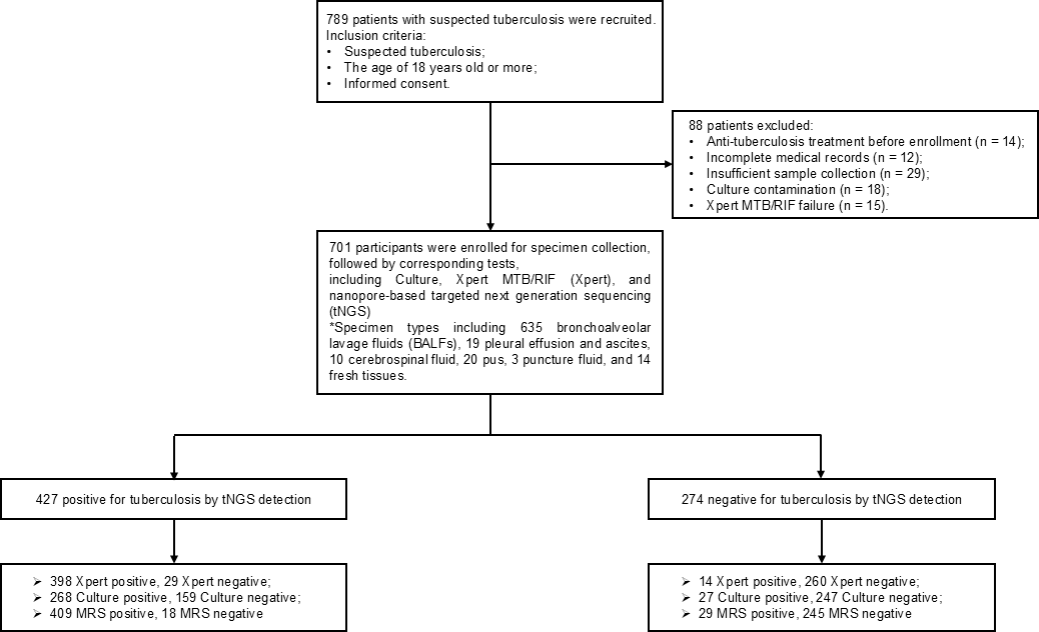
The flowchart of participant enrollment.

This study was a prospective multicenter diagnostic accuracy study performed across five TB-designated hospitals in both northern and southern China (Heilongjiang, Beijing, Chongqing, Hangzhou, and Guangzhou). From December 2023 to June 2024, non-sputum specimens were collected from 701 adult patients (aged ≥18 years) with clinical indicators suggestive of active tuberculosis. The patient inclusion criteria were as follows: (i) presence of TB-suspected symptoms, including fever, chest pain, chest tightness, cough, expectoration, or hemoptysis; (ii) chest X-ray or computed tomography findings indicative of pulmonary lesion, such as cavities, masses, scattered patchy ground-glass opacities, bronchiectasis, nodules, pulmonary plaques, bronchial spread, or other specific patterns (e.g., “hot bath” lungs, solitary nodules). Protocols for this lab-developed assay referred to the Standards for Reporting of Diagnostic Accuracy Studies guidelines (www.equator-network.org/reporting-guidelines/stard) for assessment of diagnostic accuracy. Inclusion and exclusion criteria are detailed in Fig. 1.

Participants with positive results in at least one of the tests, including culture and Xpert, were diagnosed as TB patients under the microbiological reference standard (MRS) following the Diagnosis for Pulmonary Tuberculosis Guidelines. Clinical and laboratory staff were blinded to the TB status of patients during specimen collection and processing, and participant identification numbers were restricted to the research team.

### Sample collection

Non-sputum specimen types included bronchoalveolar lavage fluids (BALFs), pleural effusion or ascites, cerebrospinal fluids, pus, puncture fluids, and fresh tissues. After enrollment, for patients highly suspected of pulmonary tuberculosis or tuberculous pleuritis, more than 4 mL of BALF or two portions of pleural tissue, with each portion approximately mung bean/pea-sized (3–5 mm in diameter), were, respectively, collected from corresponding participants. For patients highly suspected of extrapulmonary tuberculosis, samples were collected as follows: no less than 2 mL of cerebrospinal fluid from patients with tuberculous meningitis or pulmonary tuberculosis complicated by tuberculous meningitis, approximately 3 mL of aspirated fluid or pus from patients with lymph node tuberculosis, and more than 3 mL of joint pus from patients with bone tuberculosis. All samples were stored at 4°C and processed within 24 h.

All non-sputum specimens underwent standardized pretreatment prior to culture, Xpert testing, and tNGS assay. The viscous samples, including BALFs, pus, pleural effusion or ascites, and puncture fluids, were liquefied by mixing with an equal volume of liquefaction buffer, followed by adding 20 μL of Exogenous Internal Standard (EIS) reagent, thorough vortexing, incubation at 37°C for 10 min, and centrifugation at 12,000 rpm for 2 min to obtain a 30–40 μL pellet. Tissue specimens were finely minced and homogenized in a small volume of sterile phosphate buffer saline (PBS) using a tissue disruptor, then mixed with 20 μL of EIS reagent and centrifuged. Low-viscosity fluids, including cerebrospinal fluid, were directly mixed with EIS reagent and homogenized. After centrifugation, the pellets were divided into three portions for mycobacterial culture and phenotypic drug susceptibility testing (pDST), Xpert MTB/RIF testing, and nucleic acid extraction and sequencing. Nucleic acid extraction and sequencing analysis were performed by Hangzhou DIAN Biotechnology Co., Ltd. within 48 h of sample collection. In addition, clinical data were collected, and follow-ups were conducted after sample collection.

### Microbiological reference standard tests (culture and Xpert)

Processed pellets were treated with N-acetyl-L-cysteine and sodium hydroxide for 10 min, centrifuged, and resuspended in 2.5 mL PBS (16). A volume of 0.5 mL of the sediment was inoculated into a mycobacterial growth indicator tube (MGIT, Becton Dickinson Diagnostics; Franklin Lakes, NJ) after washing with PBS, then incubated for up to 6 weeks. MGIT-positive cultures were confirmed for the presence of MTB using the Capilia TB MPT64 Rapid test (Genesis, Hangzhou, China).

For the Xpert MTB/RIF assay, 1.0 mL of raw sample was mixed with 2.0 mL of sample reagent according to the manufacturer’s instructions (17). After incubation for 15 min, 2.0 mL of this mixture was pipetted into an Xpert MTB/RIF assay cartridge, which was then loaded onto a GeneXpert instrument. All remaining testing steps were performed following the manufacturer’s guidelines (18).

### Phenotypic drug susceptibility testing

Phenotypic DSTs for anti-TB drugs, including rifampicin, isoniazid, ethambutol, streptomycin, levofloxacin, amikacin, and capreomycin, were performed on all positive cultures using Thermo Scientific Sensititre *Mycobacterium tuberculosis* MYCOTBI AST Plate. The bacterial solution was added to the 96-well plate according to the instructions. After sealing the plate with a sealing film, it was incubated at 37°C for 7 days, after which the results were observed. The final concentration of the drugs was 0.2 μg/mL for isoniazid, 1 μg/mL for rifampicin, 2 μg/mL for streptomycin, 5 μg/mL for ethambutol, 2 μg/mL for levofloxacin, 4 μg/mL for amikacin, and 2 μg/mL for capreomycin (19).

### Targeted next-generation sequencing on nanopore platform

Sample preparation, DNA extraction, multiplex targeted PCR, sequencing, and bioinformatic analysis on a nanopore platform were performed as described in a previous study (20). Briefly, liquefied BALF, homogenized pleural tissue, and other samples underwent DNA extraction using the VT02-B microbial DNA extraction kit (GENEDIAN, China) according to the manufacturer’s protocol. Samples were lysed chemically, and when necessary (e.g., tissue or viscous specimens), mechanically homogenized to ensure adequate disruption. DNA was then purified through a silica membrane with standard washing and elution steps. The concentration of extracted DNA was determined using a Qubit double-stranded DNA high-sensitivity assay kit (Q32854, Thermo Fisher Scientific Inc., USA). Single-tube multiplex PCR amplification was performed to enrich the targeted gene sequences, including (i) a panel of genes covering 49 mycobacteria and 31 other pathogens, including *IS6110*, *hsp65*, 16S rRNA gene, ITS (Internal Transcribed Spacer) gene, and species-specific *gyrA* (21); (ii) 19 drug resistance-associated genes across MTB, including *rpoB*, *katG*, *inhA*, *ahpC*, *embB*, *embA*, *rpsL*, *rrs*, *pncA*, *gyrA*, *gyrB*, *rplC*, *mmpR*, *ddn*, *alr*, *eis*, *thyA*, *folC*, and *ethA* (20). Then a single-tube multiplex PCR was performed for simultaneous amplification of drug resistance-associated genes. DNA library construction involved purification, end repair, barcode ligation, re-purification, and adapter ligation. According to the manufacturer’s instructions provided in the SQK-NBD114.96 Native Barcoding Kit (Oxford Nanopore Technologies, Oxford, UK), the DNA library was loaded onto the flow cell R10.4.1 (FLO-PRO114M, ONT) and sequenced on a GridION (Nanopore) Sequencer to generate ~50 M reads per sample. To identify background microorganisms and measure detection normality, negative controls and positive controls were all established for each batch of experiments. Sequencing was carried out in batches of ~24 samples, with each sample yielding an average of ~50 million reads; thus, total reads per sequencing library were approximately 50 million × N (where *N* = number of samples per batch).

### Bioinformatics analysis

All samples were sequenced on the GridION platform (Oxford Nanopore Technologies, Oxford, UK) using the R10.4.1 flow cell, and all downstream analyses—including basecalling with Dorado, quality filtering, human read removal, microbial alignment, and resistance mutation calling—were performed using the same standardized computational pipeline for each batch of samples. During sequencing, real-time data acquisition was conducted with MinKNOW software. The current signal generated as nucleic acid molecules passed through the nanopore was continuously recorded in FAST5 format, which was then converted into raw sequence data using the Dorado and stored in FASTQ format. Initial quality control was implemented through NanoFilt with a Phred score threshold of Q9. Human sequences with identity ≥90% and coverage ≥40 were identified by mapping clean data to human reference genomes (GRCh38) and removed using Minimap2. Our in-house microbial genome database consisted of MTB, 48 non-tuberculous mycobacteria (NTM), and 31 other pathogens. And our in-house mutation database included 19 drug resistance-associated genes across MTB. To construct the database, we constructed a list according to a previous study (20). After filtering human reads, the remaining sequences were aligned to the microbial genome database, in-house scripts were employed to process the mapped data, enabling the identification of microorganisms in the sample. Finally, BLAST comparison was conducted, and non-specific reads were carefully removed through a correction procedure. For MTB positive samples, the corresponding sequences were aligned to the drug resistance-associated mutation database, and in-house scripts were employed to identify the drug resistance-associated mutations. The nanopore sequencing workflow achieved a total turnaround time of approximately 8 h from test initiation to result reporting.

### Statistical analysis

In this study, we utilized a confusion matrix to determine the performance metrics of tNGS testing, including the sensitivity, specificity, positive predictive value, and negative predictive value of tNGS testing, using the Xpert results and MRS results as diagnostic standards. To detect significant differences in copy number levels among groups, the one-way ANOVA was applied, followed by Dunn’s correction for multiple comparisons. Data visualization tasks, encompassing heatmaps, plots generated with ggplot2, and Venn diagrams, were executed using R software (version 4.3.1). Moreover, GraphPad Prism (version 8.0.1) facilitated the analysis and visualization of pathogen types and gene mutations. The differences were considered to be statistically significant at *P* <0.05.

## RESULTS

### Participant enrollment and clinical characteristics

From 22 December 2023 to 22 June 2024, we enrolled 701 individuals meeting the inclusion criteria from five different regions in China for the acquisition of non-sputum specimens for this study (Fig. 1). The mean age was 48.7 years, and 62.8% (440/701) were male. Among these participants, 635 (90.6%) provided BALF specimens, and 541 (77.2%) had one or more typical TB clinical symptoms, including fever (19.4%) and cough (66.3%). Culture and Xpert tests showed the detection rate of 42.1% and 58.8% for MTB. Respectively, tNGS yielded the highest MTB detection rate of 60.9%. In 427 tNGS positive individuals, 6.8% (29/427) were Xpert negative. And 14 individuals with tNGS negative results were confirmed as Xpert positive. Based on MRS in combination with culture and Xpert, 438 (62.5%) participants were diagnosed with active TB. The detailed information about these participants was summarized in Table 1.

**TABLE 1:** Participants’ demographic and clinical characteristics and tuberculosis detection results by different methods (*n* = 701)^*b*^

Characteristics		Total (*n* = 701)	Type of samples		
			BALF (*n* = 635)	Pleural effusion and ascites (*n* = 19)	Others^*a*^ (*n* = 47)
Sex, n (%)	Female	261 (37.2)	238 (37.5)	8 (42.1)	15 (31.9)
	Male	440 (62.8)	397 (62.5)	11 (57.9)	32 (68.1)
Age (years)	Median ± SD	48.7 ± 16.8	18.4 ± 16.8	60.4 ± 13.4	47.9 ± 15.9
Clinical symptoms (n, %)	Fever	136 (19.4)	123 (19.4)	3 (15.8)	10 (21.3)
	Cough	465 (66.3)	439 (69.1)	16 (84.2)	10 (21.3)
	Hemoptysis	66 (9.4)	64 (10.1)	2 (10.5)	0 (0.0)
	Sleep hyperhidrosis	25 (3.6)	24 (3.8)	0 (0.0)	1 (2.1)
	Fatigue/inappetence	33 (4.7)	29 (4.6)	2 (10.5)	2 (4.3)
	Pectoralgia	76 (10.8)	69 (10.9)	2 (10.5)	5 (10.6)
	Dyspnea	70 (10.0)	63 (9.9)	3 (15.8)	4 (8.5)
*Mtb* detection (n, %)	Culture positive	290 (41.4)	258 (40.6)	1 (5.3)	31 (66.0)
	Xpert MTB/RIF positive	412 (58.8)	370 (58.3)	5 (26.3)	37 (78.7)
	tNGS positive	427 (60.9)	385 (60.6)	5 (26.3)	37 (78.7)
	Microbiological reference standard	438 (62.5)	393 (61.9)	5 (26.3)	40 (85.1)

^*a*^ Others include 10 cerebrospinal fluid samples, 20 pus specimens, 3 puncture fluid samples, and 14 fresh tissues.

^*b*^ tNGS, targeted next-generation sequencing assay; BALF, bronchoalveolar lavage fluid; SD, standard deviation.

### Diagnostic performance of tNGS for active TB

Compared with Xpert and culture tests, tNGS showed a sensitivity of 96.6% (95% CI, 95.3%–97.9%) and 90.8% (95% CI, 88.7%–93.0%) and specificity of 90.0% (95% CI, 87.7%–92.2%) and 60.8% (95% CI, 57.2%–64.5%), respectively (Table 2). Based on MRS as a standard reference, the sensitivity and specificity of tNGS were 93.4% (95% CI, 91.5%–95.2%) and 93.2% (95% CI, 91.3%–95.0%). Notably, of 18 individuals who were MRS negative but tNGS positive, 15 (83.3%) were clinically diagnosed as TB patients. Given that the identification of asymptomatic TB patients remains a challenge in clinical practice, we compared the diagnostic accuracy of tNGS in symptomatic and asymptomatic populations. Utilizing MRS as a reference, the tNGS assay showed similar sensitivity (92.9% vs 94.9%) and specificity (94.0% vs 90.3%) for symptomatic and asymptomatic populations (Table S1). When stratified to various specimen types, BALF specimens had a sensitivity of 93.4% and specificity of 92.6% in comparison to MRS (Table S1). Besides, tNGS derived from pleural effusion and ascites, cerebrospinal fluid, pus, puncture fluid, and fresh tissue samples showed the sensitivity of 100.0%, 83.3%, 100.0%, 100.0%, and 92.9%, respectively. Furthermore, we also analyzed the relationship between Xpert semiquantitative grades and tNGS read counts. As shown in Fig. S1, tNGS results exhibited greater copy numbers in specimens with higher Xpert grade levels, which illustrated that tNGS data showed a positive correlation with bacterial loads in specimens.

**TABLE 2:** Diagnostic performance of tNGS when compared with Xpert MTB/RIF (Xpert), culture, and MRS^*a*^

	TP	FP	FN	TN	Sensitivity (%, 95% CI)	Specificity (%, 95% CI)	PPV (%, 95% CI)	NPV (%, 95% CI)	Overall concordance (%, 95% CI)
Xpert	398	29	14	260	96.6 (95.3–97.9)	90.0 (87.7–92.2)	93.2 (91.3–95.1)	94.9 (93.3–96.5)	93.9 (92.1–95.6)
Culture	268	159	27	247	90.8 (88.7–93.0)	60.8 (57.2–64.5)	62.8 (59.2–66.3)	90.1 (87.9–92.4)	73.5 (70.2–76.7)
MRS	409	18	29	245	93.4 (91.5–95.2)	93.2 (91.3–95.0)	95.8 (94.3–97.3)	89.4 (87.1–91.7)	93.3 (91.4–95.1)

^*a*^ TP, true positive; TN, true negative; FP, false positive; FN, false negative; PPV, positive predictive value; NPV, negative predictive value; CI, confidence interval.

### Detection of MTB and other coinfecting respiratory pathogens with tNGS

Given that the tNGS assay produces the detection results of NTM and other pathogens, we first analyzed the coexistence of MTB and NTM. In 427 MTB-positive individuals, approximately 5.2% (22/427) were coinfected with NTM (Fig. S2), such as *Mycobacterium abscessus complex* and *Mycobacterium intracellulare*. Besides, in 21 NTM-positive but MTB-negative individuals, 4 were clinically diagnosed as NTM patients. Furthermore, we also found that a proportion of TB patients were coinfected with other respiratory pathogens. The most frequent bacteria included *Klebsiella pneumoniae* ‌and *Haemophilus influenzae*, whether the presence of these pathogens affects the prognosis of TB patients requires further investigation.

### Diagnostic accuracy of tNGS for drug resistance

**Fig 2:**
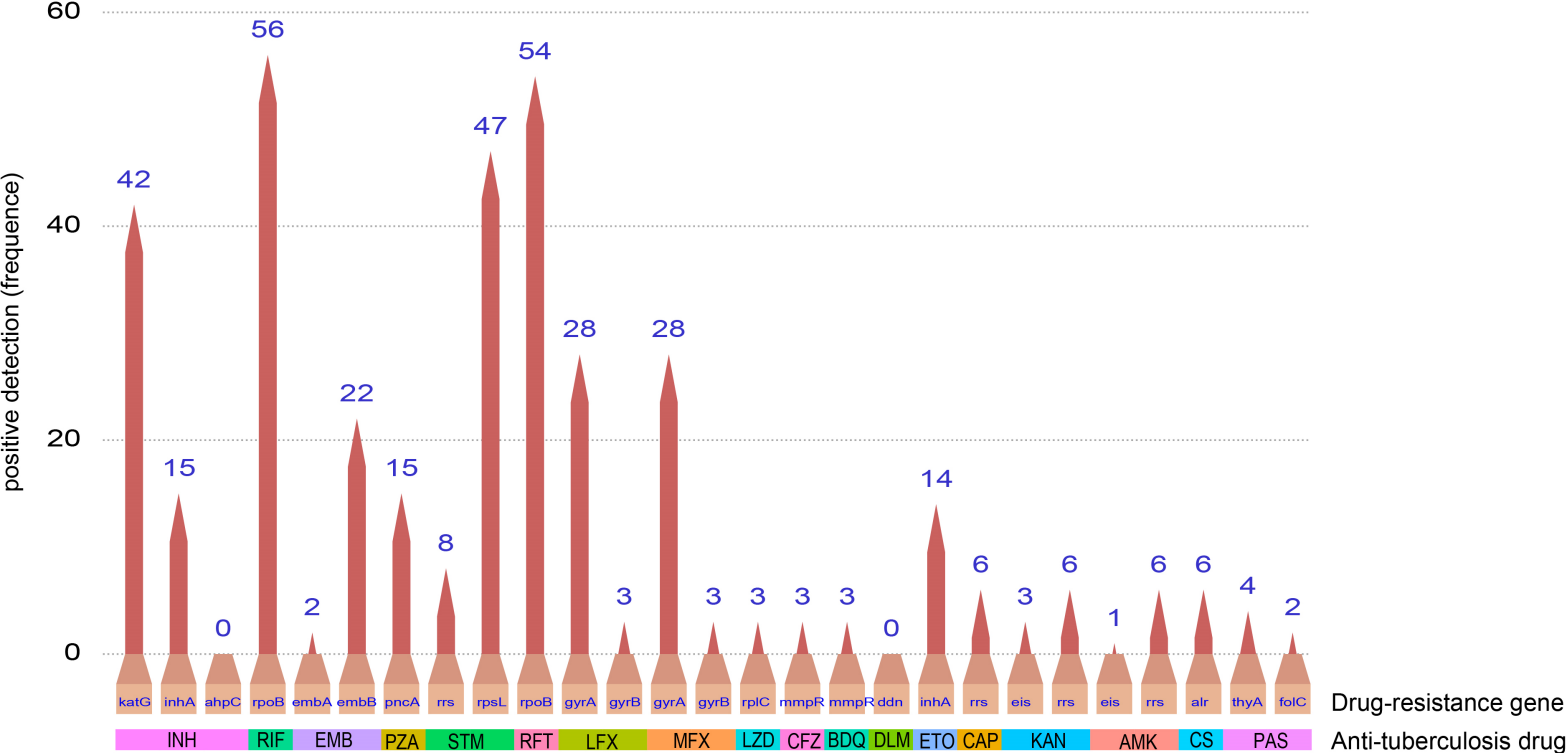
Detection of the drug resistance mutations using tNGS testing. The pencil bar chart shows the total frequencies of each drug resistance mutation. INH, isoniazid; RIF, rifampicin; EMB, ethambutol; PZA, pyrazinamide; STM, streptomycin; RFT, rifapentine; LFX, levofloxacin; MFX, moxifloxacin; LZD, linezolid; CFZ, clofazimine; BDQ, bedaquiline; DLM, delamanid; ETO, ethionamide; CAP, capreomycin; KAN, kanamycin; AMK, amikacin; CS, cycloserine; PAS, aminosalicylic acid.

**Fig 3:**
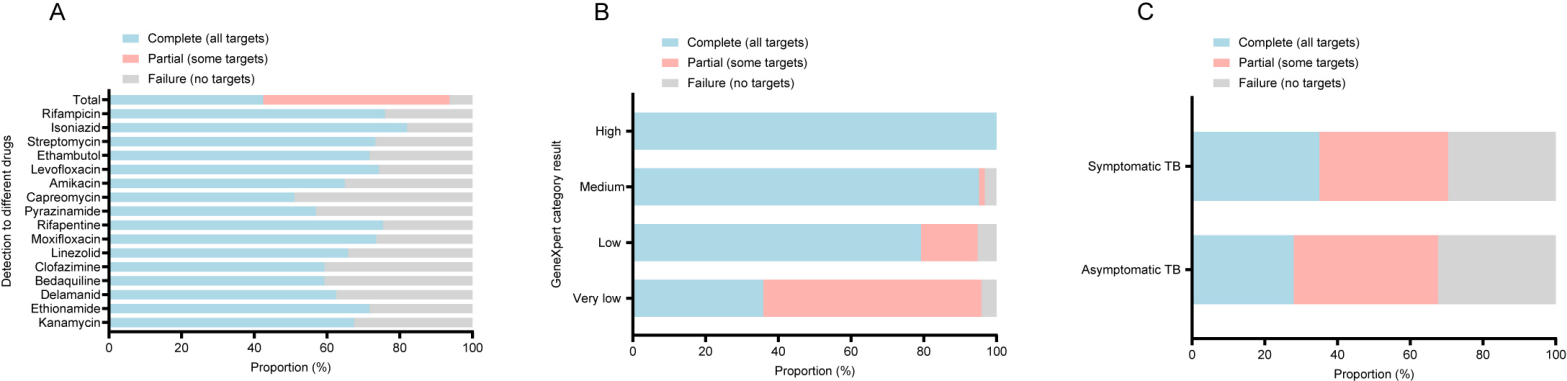
Sequencing success by tNGS-based genotypic drug susceptibility testing (different anti-TB drugs, study GeneXpert semiquantitative result category, and clinical status). (**A**) Sequencing success conditions in different anti-TB drugs. (**B**) Sequencing success conditions when stratified by different GeneXpert semiquantitative result categories. (**C**) Sequencing success conditions in the specimens derived from symptomatic and asymptomatic TB patients. Blue bars indicate the number of samples for tNGS method with complete resistance profiles (i.e., all targets were sequenced and reported). Pink bars indicate the number of samples for tNGS method with partial resistance profiles (i.e., some targets failed to be sequenced). Gray bars indicated the number of samples without resistance data generated (i.e., no targets were reported by tNGS). Asymptomatic TB patients refer to individuals who did not exhibit any TB-associated symptoms (fever, cough, hemoptysis, night sweats, chest pain, and dyspnea) but were suggestive of TB during screening.

In this study, we further assessed the resistant mutations for 18 antituberculosis drugs via tNGS assay. The frequencies of drug-resistant mutations corresponding to each drug were visualized in Fig. 2 and Fig. S3. The highest frequencies of mutation were noted in the katG gene for isoniazid (42/427) resistance, rpoB gene for rifampicin (56/427) and rifapentine (54/427) resistance, and rpsL gene for streptomycin (47/427) resistance. In 427 individuals whose tNGS results were positive, 93.7% (400/427) received complete or partial drug resistance profiles (Fig. 3A). When stratified by Xpert semiquantitative grades, tNGS showed superior complete detection rate in specimens with higher Xpert grade levels, from 100.0% in the “high” category to 35.9% in the “very low” category, which illustrated that the detection of drug resistance by tNGS was dependent on bacterial load (Fig. 3B). Furthermore, 70.4% of symptomatic TB patients and 67.6% of asymptomatic TB patients received complete or partial drug resistance results through tNGS (Fig. 3C).

Of 427 tNGS positive individuals enrolled in the present study, 268 participants had culture-positive specimens and further phenotypic DST results. Based on phenotypic DST as a reference standard, we evaluated the accuracy of tNGS to detect the drug resistance. In 268 non-sputum specimens, tNGS results showed high concordance with phenotypic DST, ranging from 94.8% (254/268, streptomycin) to 98.9% (265/268, rifampicin and amikacin) (Table 3). As shown in Table 3, the sensitivity and specificity of tNGS compared with phenotypic DST were 92.6% (95% CI, 79.8%–98.1%) and 100% (95% CI, 97.9%–100.0%) for rifampicin, 88.2% (95% CI, 71.6%–96.1%) and 98.7% (95% CI, 96.0%–99.7%) for isoniazid, 85.4% (95% CI, 70.1%–93.9%) and 96.5% (95% CI, 92.9%–98.4%) for streptomycin, 82.4% (95% CI, 56.0%–95.3%) and 98.8% (95% CI, 96.3%–99.7%) for ethambutol, 86.2% (95% CI, 67.4%–95.5%) and 99.6% (95% CI, 97.3%–100.0%) for levofloxacin, 66.7% (95% CI, 24.1%–94.0%) and 99.6% (95% CI, 97.6%–100.0%) for amikacin, and 50.0% (95% CI, 9.2%–90.8%) and 98.9% (95% CI, 96.4%–99.7%) for capreomycin, respectively.

**TABLE 3:** Detection performance of tNGS for drug-resistant *Mtb* compared with phenotypic drug susceptibility test^*a*^

Drug		TP	FP	FN	TN	Sensitivity (%, 95% CI)	Specificity (%, 95% CI)
Rifampicin	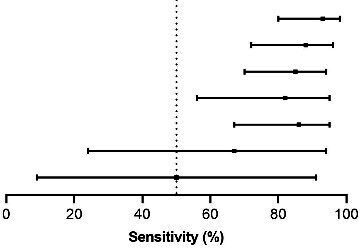	38	0	3	227	92.6 (79.0–98.1)	100.0 (97.9–100.0)
Isoniazid		30	3	4	231	88.2 (71.6–96.1)	98.7 (96.0–99.7)
Streptomycin		35	8	6	219	85.4 (70.1–93.9)	96.5 (92.9–98.4)
Ethambutol		14	3	3	248	82.4 (56.0–95.3)	98.8 (96.3–99.7)
Levofloxacin		25	1	4	238	86.2 (67.4–95.5)	99.6 (97.3–100.0)
Amikacin		4	1	2	263	66.7 (24.1–94.0)	99.6 (97.6–100.0)
Capreomycin		2	3	2	261	50.0 (9.2–90.8)	98.9 (96.4–99.7)

^*a*^ Sensitivities of tNGS for different anti-TB drugs are shown in the forest plot diagrams, while both sensitivities and specificities are shown in the adjacent tables. For anti-TB drugs, MGIT phenotypic drug susceptibility tests were used as the gold standard. The dotted line represents the sensitivity with 50%. TP, true positive; FP, false positive; FN, false negative; TN, true negative; CI, confidence interval.

## DISCUSSION

The scale-up of WHO-recommended rapid diagnostics (WRDs) for direct use on sputum has significantly contributed to the global increase in TB case detection (6). Unfortunately, current diagnostic algorithms fail to detect tubercle bacilli in approximately half of reported TB cases. There is therefore a great need for ultra-sensitive tests that can accurately identify clinically diagnosed TB patients (22). In the present study, we prospectively assessed the performance of tNGS-based assay on the diagnosis of active TB cases. Results from this multicenter study demonstrated that tNGS exhibited superior sensitivity and specificity compared to standard Xpert and MGIT methods for detecting TB cases, particularly in participants with low bacterial loads, regardless of the reference standard used. Additionally, the tNGS assay showed high sensitivity for TB case detection in patients with extrapulmonary TB. Our findings were consistent with multiple previous results demonstrating that tNGS yielded robust accuracy compared with Xpert assay and MRS (23–25). In clinical practice, the performance of tNGS could facilitate early diagnosis of various forms of TB, especially in patients with TB with low bacterial loads.

Recently, the WHO adopted the term “asymptomatic TB” to better recognize individuals with TB disease who do not report symptoms suggestive of active TB (26). A recent meta-analysis of TB prevalence surveys found that approximately half of all TB cases were completely asymptomatic (27). Individuals with asymptomatic TB are expected to have a lower bacterial burden compared to those with symptomatic TB (28, 29), presenting particular challenges for laboratory diagnosis using commercially available technologies. Our study demonstrated that the tNGS-based assay showed high diagnostic accuracy in asymptomatic TB individuals, indicating that the majority of asymptomatic TB cases were accurately identified, thereby minimizing unnecessary evaluations. However, the invasive procedures of bronchoalveolar lavage constitute barriers to effective scale-up in the community-based active case-finding interventions (30). Further evaluation of this assay on tongue swab is of great importance to provide an alternative diagnostic approach for community-based screening (29).

Previous studies confirmed that patients coinfected with MTB and NTM were usually associated with poor outcomes compared with single MTB infection (31–33), partly due to the failure in identifying the coexistence of the mycobacterial species simultaneously. In our study, tNGS-based results revealed an approximate 5% NTM coinfection rate among MTB-positive individuals, suggesting that the tNGS assay is a promising method for the simultaneous detection of MTB and NTM (34). Additionally, we also observed that other respiratory pathogens were reported in 5.2% TB patients. An interesting question arises regarding whether the presence of these additional pathogens is associated with clinical symptoms and treatment outcomes in TB patients. Further studies are needed to elucidate the potential host–pathogen interactions in these individuals. Understanding these interactions could provide valuable insights into improving diagnostic methods and therapeutic strategies for TB patients with complex coinfections.

There is clearly a great need for rapid tests that can simultaneously detect multiple drug resistance of MTB (35). The recent development of portable nanopore sequencing instruments accelerates the clinical diagnosis of drug-resistant TB in a cost- and time-effective manner (10). In a recent prospective evaluation of commercial tNGS assays, 91% of molecularly confirmed pulmonary TB patients produced adequate tNGS sequencing data, at least partial drug resistance profiles (36), which was slightly lower than 93.3% with our sequencing panel. This difference may be partly attributed to the optimized lysis procedures in DNA extraction, resulting in higher DNA yield. Our data also imply that the limits of detection (LODs) of the tNGS assay for drug resistance can be improved further with revisions to the targeted gene panel. Generally, the detection failure in commercial tNGS assays appears to be primarily caused by bacterial load in the clinical samples, which only allows it as a subsequent test for patients with positive Xpert results (36). However, the higher sequencing success rate of our diagnostic panel highlights the potential for using the tNGS assay as an initial diagnostic test for suspected drug-resistant TB patients, thereby offering the prospect of personalized care for multidrug-resistant (MDR) or rifampicin-resistant (RR) TB.

Another important finding of the present study was the emerging bedaquiline (BDQ) resistance in the Chinese population, 4/252 (1.6%) of whom harbored resistance-associated mutations. A prospective *in vitro* surveillance study in China revealed that the overall resistance rate of MTB isolates to BDQ was 0.2% in 2022, which was dramatically lower than our observation (37). Specifically, the poor correlation between mutations and phenotypic BDQ resistance leads to an underestimation of the prevalence of BDQ resistance in our cohort. The increasing trend of BDQ resistance undoubtedly impairs the efficacy of WHO-endorsed regimens in patients affected by MDR/RR-TB, emphasizing that routine surveillance of BDQ resistance is critical for accurate planning and formulation of TB control activities (38).

This study has several limitations. First, the study was powered to evaluate the accuracy of the tNGS assay in the detection of MTB and drug resistance simultaneously, resulting in large confidence intervals when examining the drug resistance due to the small sample size in drug-resistant TB patients. Second, the majority of non-sputum specimens were collected by bronchoalveolar lavage, which may limit the generalizability of our conclusions to extrapulmonary TB specimens. Third, the Xpert assay, rather than next-generation Xpert Ultra, was used as a reference method in our evaluation due to accessibility constraints during the study period. Considering that Xpert Ultra is substantially more sensitive than Xpert for the detection of TB with low bacterial loads, the specificity of tNGS assay may be underestimated when compared to a microbiological reference standard. Fourth, *in vitro* susceptibility testing for the new and repurposed anti-TB drugs was not performed, precluding an evaluation of the tNGS assay’s performance in detecting resistance to these agents. Fifth, although the assay was designed to detect identification targets at ~10–50 copies/mL and resistance genes at ~50–100 copies/mL, a formal analytical LOD was not experimentally established in this study. Nevertheless, our study provides new evidence for the clinical application of the tNGS assay on non-sputum specimens from TB patients with low bacterial loads.

In summary, the tNGS assay is a rapid and highly sensitive tool for TB case detection and simultaneous detection of drug resistance in non-sputum specimens. Its sensitivity advantage over conventional methods is most pronounced in TB patients with low bacterial loads. Correspondingly, the high sensitivity of the tNGS assay also raises concern about its specificity in identifying TB cases. Further studies are urgently required to optimize the cut-off value for the tNGS assay to balance the sensitivity and specificity stratified to diagnostic or screening purposes. Optimizing these parameters will enhance the clinical utility of the tNGS assay and improve its accuracy in diverse settings, ultimately leading to better patient management and public health strategies for TB control.

## Data Availability

The raw sequence data reported in this paper have been deposited in the Genome Sequence Archive (39) in the National Genomics Data Center (40), China National Center for Bioinformation/Beijing Institute of Genomics, Chinese Academy of Sciences (GSA-Human: HRA016472) that are publicly accessible at https://ngdc.cncb.ac.cn/gsa-human.

## SUPPLEMENTAL MATERIAL

Supplemental material jcm.01433-25-s0001.docx Tables S1 and S2; Fig. S1 to S3.

Graphical abstract jcm.01433-25-s0002.tiff Visual depiction of the study.

ASM does not own the copyrights to Supplemental Material that may be linked to, or accessed through, an article. The authors have granted ASM a non-exclusive, world-wide license to publish the Supplemental Material files. Please contact the corresponding author directly for reuse.
