# Supplementary material for: Accuracy of nanopore-based targeted next-generation sequencing assay for detection of Mycobacterium tuberculosis and drug resistance from non-sputum specimens: a multicenter prospective study in China
Source: J Clin Microbiol. 2026 Feb 24;64(4):e01433-25. doi: 10.1128/jcm.01433-25 (PMC13059801; doi:10.1128/jcm.01433-25)
Supplement: Supplemental material — Tables S1 and S2; Fig. S1 to S3. [file jcm.01433-25-s0001.docx]

**Table S1. Diagnostic accuracy of tNGS when compared with Xpert MTB/RIF (Xpert), culture, and microbiological reference standard (MRS) when stratified by clinical symptoms.**

|  | Symptomatic patients (n = 541) | | | | Asymptomatic patients* (n = 160) | | | |
| --- | --- | --- | --- | --- | --- | --- | --- | --- |
|  | Sensitivity  (%, 95% CI) | Specificity  (%, 95% CI) | PPV  (%, 95% CI) | NPV  (%, 95% CI) | Sensitivity  (%, 95% CI) | Specificity  (%, 95% CI) | PPV  (%, 95% CI) | NPV  (%, 95% CI) |
| Xpert | 96.6  (95.0-98.1) | 91.0  (88.6-93.4) | 93.9  (91.9-95.9) | 94.8  (93.0-96.7) | 96.8  (94.0-99.5) | 86.6  (81.2-91.9) | 90.9  (86.5-95.4) | 95.1  (91.7-98.4) |
| Culture | 90.8  (88.4-93.3) | 61.5  (57.4-65.6) | 63.4  (59.4-67.5) | 90.1  (87.6-92.7) | 90.9  (86.5-95.4) | 58.5  (50.9-66.1) | 60.6  (53.0-68.2) | 90.2  (85.5-94.8) |
| MRS | 92.9  (90.8-95.1) | 94.0  (92.0-96.0) | 96.3  (94.8-97.9) | 88.7  (86.1-91.4) | 94.9  (91.5-98.3) | 90.3  (85.7-94.9) | 93.9  (90.2-97.6) | 91.8  (87.6-96.1) |

*Asymptomatic patients refer to individuals who do not exhibit any noticeable clinical symptoms associated with TB, including fever, cough, hemoptysis, night sweats, chest pain, and dyspnea).

Abbreviations: PPV, positive predictive value; NPV, negative predictive value; CI, confidential interval.

**Table S2. Evaluation of diagnostic performance of tNGS in different specimen types compared with MRS.**

| Type of samples | | TP | FP | FN | TN | Sensitivity  (95% CI) | Specificity  (95% CI) | PPV  (%, 95% CI) | NPV  (%, 95% CI) |
| --- | --- | --- | --- | --- | --- | --- | --- | --- | --- |
| BALF | | 367 | 18 | 26 | 224 | 93.4 (91.5-95.3) | 92.6 (90.5-94.6) | 95.3 (93.7-97.0) | 89.6 (87.2-92.0) |
| Others* | Pleural effusion and ascites | 5 | 0 | 0 | 14 | 100.0 (100.0-100.0) | 100.0 (100.0-100.0) | 100.0 (100.0-100.0) | 100.0 (100.0-100.0) |
|  | Cerebrospinal fluid | 5 | 0 | 1 | 4 | 83.3 (60.2-100.0) | 100.0 (100.0-100.0) | 100.0 (100.0-100.0) | 80.0 (55.2-100.0) |
|  | Pus | 17 | 0 | 0 | 3 | 100.0 (100.0-100.0) | 100.0 (100.0-100.0) | 100.0 (100.0-100.0) | 100.0 (100.0-100.0) |
|  | Puncture fluid | 3 | 0 | 0 | 0 | 100.0 (100.0-100.0) | / | 100.0 (100.0-100.0) | / |
|  | Fresh tissues | 13 | 0 | 1 | 0 | 92.9 (79.4-100.0) | / | 100.0 (100.0-100.0) | / |
|  | Total | 43 | 0 | 2 | 21 | 93.3 (87.3-99.4) | 100.0 (100.0-100.0) | 100.0 (100.0-100.0) | 87.5 (79.5-95.5) |

*Others include 19 pleural effusion and ascites, 10 cerebrospinal fluids, 20 pus, 3 puncture fluid, and 14 fresh tissues.

Abbreviations: PPV=positive predictive value. NPV=negative predictive value. CI=confidential interval.


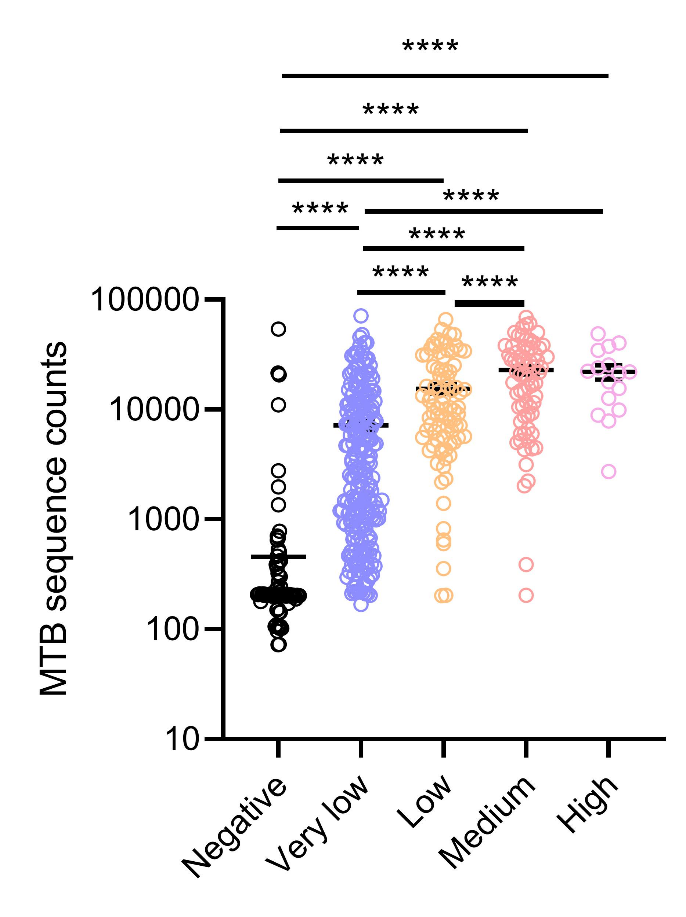


**Figure** **S1. Sequence number distribution among different GeneXpert semiquantitative result categories.**

Significant differences in copy number levels between groups were analyzed using the one-way ANOVA coupled with Dunn’s correction for multiple comparisons. Data were denoted by means with 95% CI. **** means *P* < 0.0001.


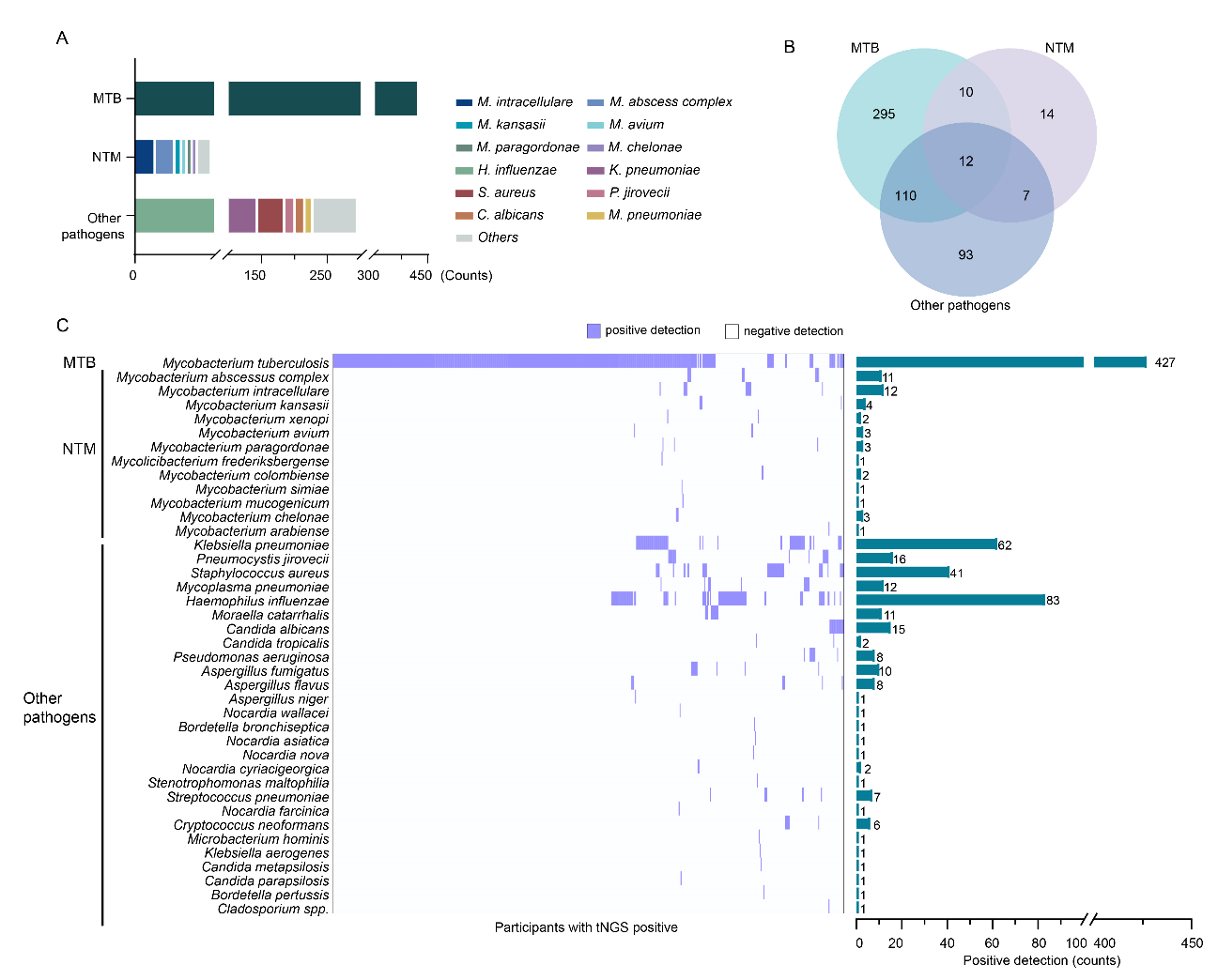


**Figure S2. Detection of MTB, NTM, and plausible pathogens using tNGS testing.**

(A) Composition and frequencies of pathogens in participants. (B) Venn diagram showing the overlapping of detection of MTB, NTM, and other plausible pathogens in participants. (C) The distribution of pathogen spectrum in each participant. *Left*, the blue blocks in heatmap represent the positive detection for specific pathogen, write blocks in heatmap represented negative detection for specific pathogen. Column charts on the *right* show the total number of each pathogen.


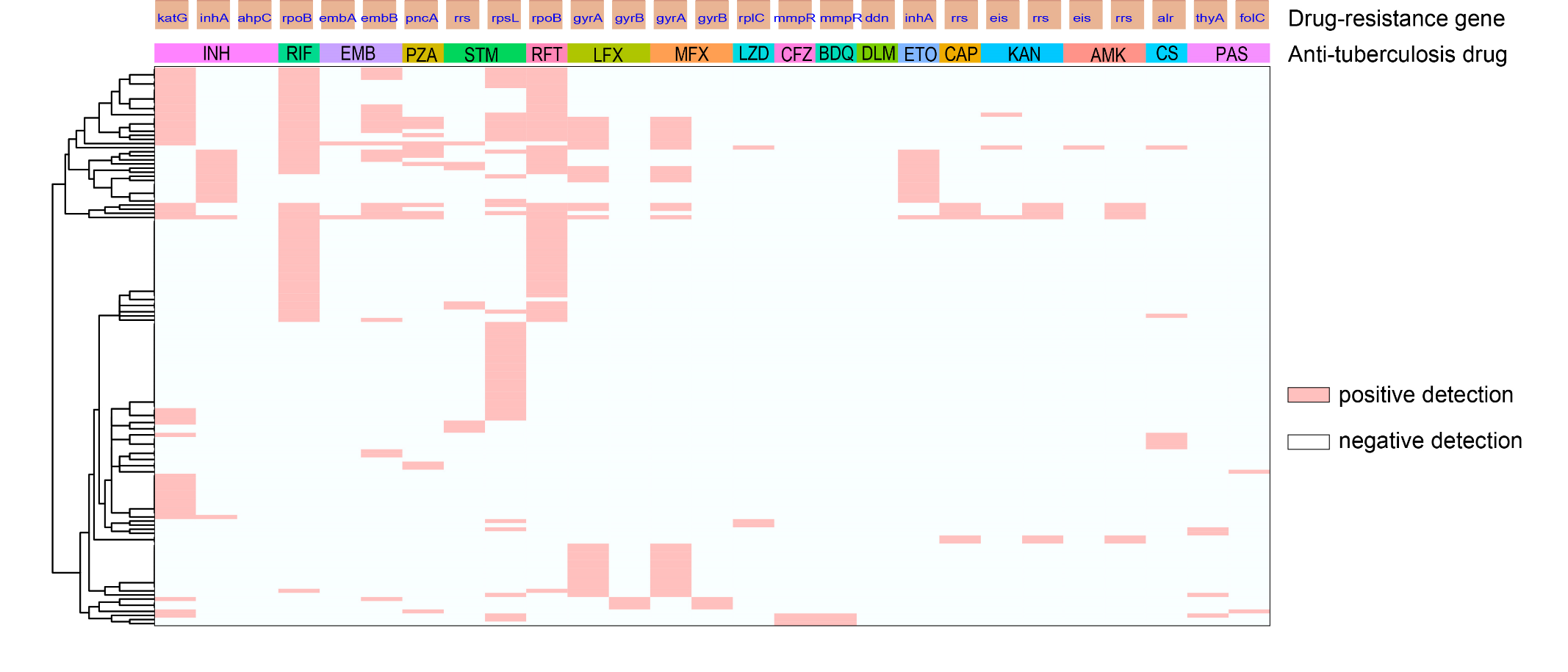


**Figure S3. Heatmap shows detailed distribution of drug-resistant mutations, including first-line, second-line and other common anti-tuberculosis drugs.**

Red blocks represent positive detection of drug resistance mutations, and white represented negative detection of drug resistance mutations.
