# Supplementary figures and images for: Accuracy of nanopore-based targeted next-generation sequencing assay for detection of Mycobacterium tuberculosis and drug resistance from non-sputum specimens: a multicenter prospective study in China
Source: J Clin Microbiol. 2026 Feb 24;64(4):e01433-25. doi: 10.1128/jcm.01433-25 (PMC13059801; doi:10.1128/jcm.01433-25)

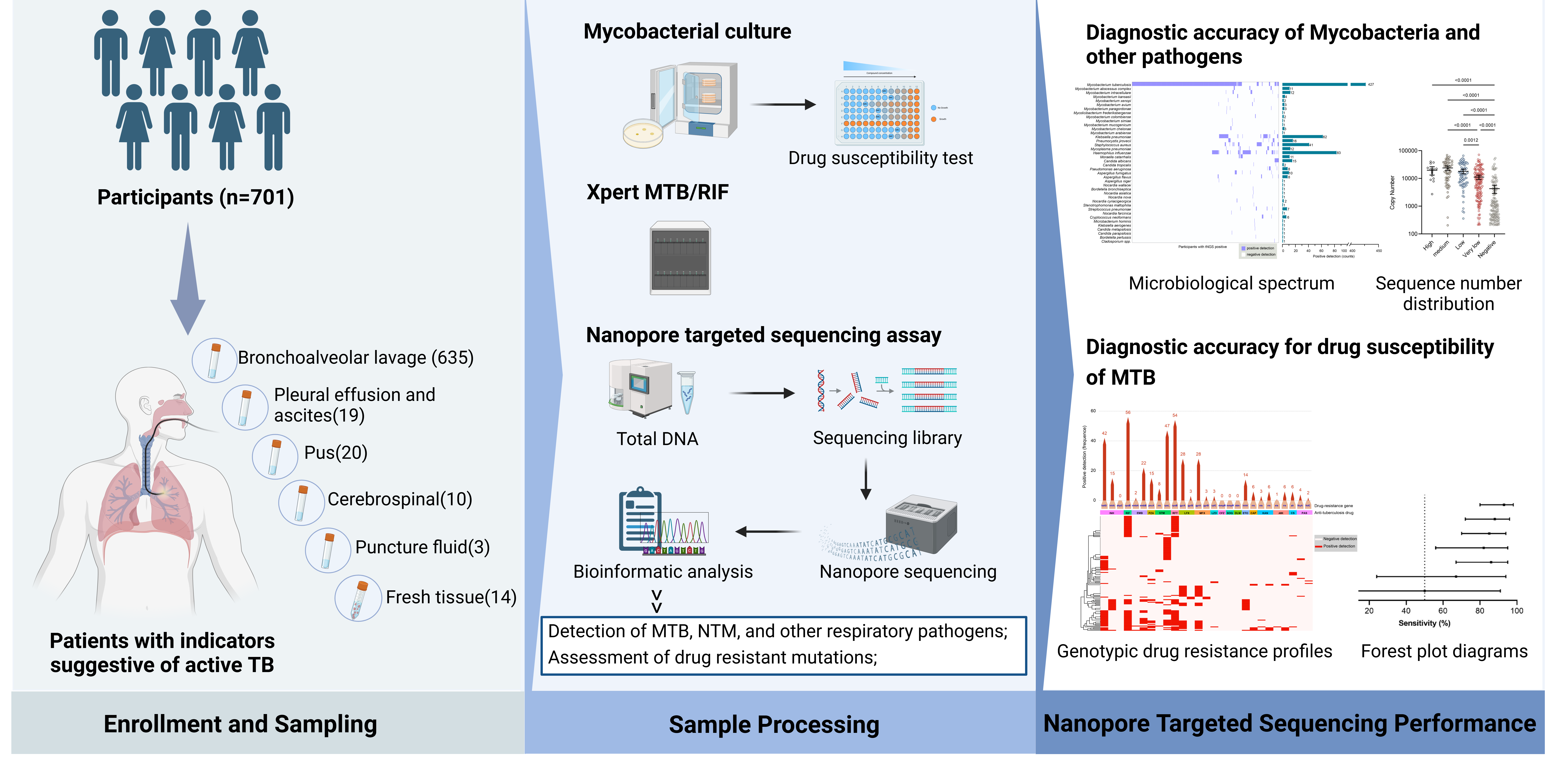

Supplement: Graphical abstract — Visual depiction of the study. [file jcm.01433-25-s0002.tiff]
